# Metabolomic changes in cats with renal disease and calcium oxalate uroliths

**DOI:** 10.1007/s11306-022-01925-4

**Published:** 2022-08-13

**Authors:** Dennis E. Jewell, Selena K. Tavener, Regina L. Hollar, Kiran S. Panickar

**Affiliations:** 1 Department of Grain Science and Industry, Kansas State University, Manhattan, KS 66506 USA; 2 Science and Technology Center, Hill’s Pet Nutrition®, Inc, Topeka, KS 66617 USA

**Keywords:** Disease markers, Kidney, Inflammation, Nephroliths

## Abstract

**Introduction:**

There is a significant incidence of cats with renal disease (RD) and calcium oxalate (CaOx) kidney uroliths in domesticated cats. Foods which aid in the management of these diseases may be enhanced through understanding the underlying metabolomic changes.

**Objective:**

Assess the metabolomic profile with a view to identifying metabolomic targets which could aid in the management of renal disease and CaOx uroliths.

**Method:**

This is a retrospective investigation of 42 cats: 19 healthy kidney controls, 11 with RD, and 12 that formed CaOx nephroliths. Cats were evaluated as adults (2 through 7 years) and at the end of life for plasma metabolomics, body composition, and markers of renal dysfunction. Kidney sections were assessed by Pizzolato stain at the end of life for detection of CaOx crystals. CaOx stone presence was also assessed by analysis of stones removed from the kidney at the end of life.

**Results:**

There were 791 metabolites identified with 91 having significant (p < 0.05, q < 0.1) changes between groups. Many changes in metabolite concentrations could be explained by the loss of renal function being most acute in the cats with RD while the cats with CaOx stones were intermediate between control and RD (e.g., urea, creatinine, pseudouridine, dimethylarginines). However, the concentrations of some metabolites differentiated RD from CaOx stone forming cats. These were either increased in the RD cats (e.g., cystathionine, dodecanedioate, 3-(3-amino-3-carboxypropyl) uridine, 5-methyl-2′-deoxycytidine) or comparatively increased in the CaOx stone forming cats (phenylpyruvate, 4-hydroxyphenylpyruvate, alpha-ketobutyrate, retinal).

**Conclusions:**

The metabolomic changes show specific metabolites which respond generally to both renal diseases while the metabolomic profile still differentiates cats with RD and cats with CaOx uroliths.

## Introduction

During the mid-1980’s there was a reported increase in CaOx stone formation of domestic cats (Osborne et al., 2009). In humans, CaOx stones are also the most prevalent stone type (Lieske, et al., 2014). The occurrence of CaOx uroliths in cats has compartively declined so that in 2003 the frequency of CaOx uroliths (44.3%) was similar to struvite (44.9%) (Osborne et al., 2009). Calcium oxalate uroliths are unlike struvite uroliths in that although there are a number of possible management options (DeFarges et al., 2013) they are not readily dissolvable (Lulich et al, 2013). There is a known connection between CaOx uroliths and renal disease (RD) (O’Kell et al, 2017). It is likely that the increased loss of renal function seen in some cats with CaOx stones is through damage from urine blockage. This may be because of an increased incidence of infection or other associated damage. Cats with CaOx uroliths were reported to have increased symmetric dimethylarginine (SDMA), creatinine, and reduced urine specific gravity (uSG) as well as having a reduced lifespan as compared to all other cats (Hall et al., 2017b).

The relationship of metabolomic changes in RD has been recently reviewed (Wang et al., 2019). In this review it was reported that RD resulted in plasma concentration changes resulting from changes in the metabolism of: energy, nucleic acids, amino acids, lipids, and carbohydrates. These data were mostly from changes in the plasma of humans with RD. Of specific interest to this study were the reported increase in arginine and dimethyl arginines, citrulline, allantoin, pseudouridine, and kynurenic acid. A previous study reported the differences in metabolites of healthy cats and cats with RD (Hall et al, 2020). The metabolites were either increased (urea, citrulline, dimethyl arginines, *N*-acetylarginine, glycine, *S*-adenosylhomocysteine) or were decreased (malate, γ-tocopherol/βtocopherol) in RD cats as compared to healthy controls. The metabolomic changes associated with CaOx stones are less reported. However, a study reporting CaOx stones in rats (Gao et al., 2016) reported 13 metabolite changes in the serum. Of specific interest are the increased concentrations of: 4-Hydroxy-l-proline, creatine, valerylglycine, and cholic acid. The metabolites that declined with stone formation were: succinic acid, l-carnitine, propionylcarnitine, 2-Methylbutyroylcarnitine, and taurocholic acid.

Some of the likely causes of RD include infectious disease (Pedersen, 2009), and renal lymphosarcoma (DiBartola et al., 1987), as well as nephritis and other inflammatory diseases (Weiss et al, 1996). Early detection of acute renal damage can facilitate intervention and reduce disease severity (Grauer, 2005). Regarding CaOx stone formation supersaturation of calcium oxalate is required, however, healthy normal cats may have urine which is supersaturated for CaOx (Hezel et al, 2007). A review of CaOx stone formation (Bartges, 2016) states that known risk factors include: increased urinary excretion of calcium or oxalate, aciduria, a reduction in urinary inhibitors of stone formation, and reduced urine volume.

Cats with RD benefit from foods with controlled phosphorus and protein (Elliott et al, 2000; Ross et al, 2006). There is also a benefit for cats with early stage renal decline (SDMA > 14 μg/dl) which was not severe enough to be RD to consume foods enhanced with dietary n-3 fatty acids and antioxidants (Hall et al., 2016). An increased water intake and foods with moderate dietary magnesium, phosphorus, or calcium concentrations have been shown to aid in the management of CaOx urolith formation (Lekcharoensuk et al., 2001). Increased dietary fatty acids which are an aid in the management of RD have also been shown to reduce the risk of feline CaOx (Hall et al, 2017a).

The hypothesis evaluated in this study is that each specific disease state will result in metabolomic changes in the plasma associated with that disease. Plasma was used to reduce the metabolite contamination of hemolysis. This metabolomic milieu as well as the markers of renal function, body composition, and circulating concentrations of calcium and phosphorus were evaluated to not only differentiate cats with these diseases but suggest targets for solutions that aid in their management.

## Materials and methods

This study was approved by the Institutional Animal Care and Use Committee, Hill’s Pet Nutrition, Inc., Topeka, KS. There were 42 cats used in this study (Table 1). All cats in the Hill’s Pet Nutrition, Inc. colony at the end of their life were available for inclusion in this study. Cats were assigned to the CaOx stone formers if during their life they had confirmed CaOx stones. Cats were assigned to the RD group if they were diagnosed with renal disease. Cats were chosen for the healthy kidney controls by attempting to match age at death of the two disease groups and having died with no perceived stones or loss of renal function.

**Table 1:** Patient characteristics at end of life for each group. Data are represented as absolute counts or as means (standard deviations)

	Overall	Healthy kidneys	Renal disease	Ca Oxalate stone formers
Number of cats	42	19	11	12
Male neutered	21	9	3	9
Female spayed	21	10	8	3
Mean age at death (years)	11.5 (3.3)	11.0 (3.1)	13.3 (4.2)	10.5 (2.2)

There were no statistically significant (p < 0.05) differences between groups in number of male or female cats

During their annual physical examination, plasma was analyzed for the reported analytes except metabolomics and SDMA. Metabolomics and SDMA concentrations were evaluated from banked samples (stored at − 80 °C). Body composition was evaluated at the end of life and during adult life by dual energy X-ray methods previously described (Toll et al., 1994). For the variables where adult cat response was calculated, the mean value of each cat averaged from analysis after 1 year of age and before 8 years of age. All cats were allowed exercise in indoor runs and had access to toys. Cats were housed with access to natural light and enclosed porches with lighting that varied with seasonal changes. In accordance to accepted veterinary practice all pets were cared for and supported during their life to optimize quality of life, even in the presence of CaOx stones or renal disease. Samples were obtained in a postmortem examination to discover the cause of death or the extent of disease. Euthanasia was performed in order to minimize the pets’ pain and suffering when it was concluded that the pets’ quality of life was degraded to a point where it was no longer humane to do otherwise.

### Metabolomics

Samples archived for metabolomic analysis were collected during annual exams or at the end of life. The Healthy Kidney group (n = 19) had 100 samples analyzed, the Renal Disease group (n = 11) had 52 samples analyzed and the CaOx group (n = 12) had 60 samples anlayzed. A commercial laboratory (Metabolon, Morrisville, NC, USA) performed the analysis as described previously (Hall et al., 2018). In brief, samples were extracted with methanol under vigorous shaking for 2 min to precipitate protein and dissociate small molecules bound to protein or trapped in the precipitated protein matrix, followed by centrifugation to recover chemically diverse metabolites. The resulting extract was divided into five fractions: two for analysis by two separate reverse phase methods using positive ion mode electrospray ionization (ESI), one for analysis by RP/UPLC-MS/MS using negative ion mode ESI, one for analysis by HILIC/UPLC-MS/MS using negative ion mode ESI, and one reserved for backup. Proprietary software was used to match ions to an in-house library of standards for metabolite identification and for metabolite quantitation by peak area integration.

### Complete blood count (CBC) and selected analytes

CBC was conducted by an in-house laboratory (Sysmex America Inc, XT-2000 v Automated Hematology Analyzer, Lincolnshire, IL USA) as were serum albumin, phosphorus, urea and creatinine (Roche Diagnostics, Cobas 6000 series, c501 module, Indianapolis, IN, USA). Also determined was urine specific gravity (uSG) using a refractometer. Analysis of symetric dimethyl arginine (SDMA) was accomplished by a commercial laboratory (IDEXX, Inc. IDEXX BioResearch Preclinical Research, three Centennial Drive, North Grafton MA 01536). Body composition was determined in-house (DXA-QDR-4500, Hologic, Inc., Waltham, MA). CaOx stones were analyzed by the Minnesota Urolith Center (University of Minnesota, St. Paul, MN, USA). Histology was prepared at Kansas State University and stained in-house using the Pizzolato staining method to determine the presence of CaOx stone formation in the kidney.

### Pizzolato staining

Staining for detecting CaOx stones in renal tissues was done using the modified method described (Pizzolato, 1964). In short, both kidneys were removed from the abdominal cavity during the necropsy, excess tissue was removed, and assessed for visual normality. Any visual abnormalities are noted at this time. Formalin-fixed samples (10% formalin) collected from necropsies were transferred to the histopathology lab at Kansas State University for further pathological processing and analysis. The paraffin-embedded blocks (FFPE) are returned to in house for further staining. The FFPE were sectioned at five microns and transferred onto slides for the Pizolatto staining procedure.

### Statistical analysis

Statistical analysis was completed using SAS 9.4 or JMP (SAS Institute, Cary, NC, USA). To test the hypothesis that classification (RD, CaOX, or healthy) was a significant component of the observed variation, statistical analysis of the metabolomic data was performed on natural log transformed data and age was used as a covariant. As it is common for metabolomic data to not have normally distributed residuals in the analysis of variance, log transformation was done in order to improve the models by reducing uniquely high or low values to produce normal or near-normal residuals. Although other transformations can be useful (such as square root or cube root transformations) the log transformation is most common and has been defined as standard practice for metabolomic analysis (Xia et al., 2013). All analytes with p < 0.05 and q < 0.1 were included in Table 2. For the non-metabolomic data, adult values were calculated by taking the mean of the values before diagnosis from 2 to 7 years of age. In both metabolomic and non-metabolomic data analysis cat identification was used as a random effect when there was multiple measures for the same response of a given cat. Age was used as a continuous variable in the metabolomic data as it was not binned between adult and end of life analysis. Classification was always used as a fixed effect. The post-hoc comparisons for the metabolomic data used Welch’s two-sample *t*-test, while in the non-metabolomic data the PDIFF option was used in PROC Mixed of SAS. Gender distribution was evaluated by the PROC Frequency procedure of SAS. A p-value < 0.05 was used as a significant cutoff for all anlaysis. In order to control the false discovery rate in the metabolomic analysis a q value of < 0.1 was used. This gives an accurate measure of the level of false positives and strikes a balance between accepting a difference that in reality only exists because of random variation and missing a difference that is real but does not rise to the pre-established level of significance. This approach to false discovery rate has been shown to be valuable (Storey, 2002) and recently reviewed in comparison to other methods of controling the false discovery rate (Cheng & Pounds, 2007). A correlation analysis was completed to evaluate the effect of gender and lean body mass on indicators of renal disease (SDMA and creatinine).

**Table 2:** Effect of renal disease or calcium oxalate stone formation on metabolite ratios^a^ in cats

Biochemical	Pubmed ID	RD/healthy	CaOx/healthy	RD/CaOx
*N*-Acetylserine	65249	**1.22**	1.08	1.13
*N*-Acetylthreonine	152204	**1.35**	**1.22**	1.1
*N*,*N*-Dimethylalanine	5488191	**2.1**	2.04	1.03
*N*-Carbamoylalanine	426409	2.88	**1.78**	1.62
*N*-Acetylasparagine	99715	*0.84*	0.93	0.9
Hydroxyasparagine	97663	**1.46**	**1.15**	1.27
1-Methylhistidine	92105	**2.09**	**1.59**	1.31
1-Methyl-5-imidazoleacetate	6451814	**4.07**	**2.19**	1.86
N6,N6-Dimethyllysine	193344	**1.24**	1.01	1.23
5-(Galactosylhydroxy)-l-lysine	123986	**1.96**	**1.31**	1.5
6-Oxopiperidine-2-carboxylate	3014237	2.28	**1.42**	1.61
*N*,*N*,*N*-Trimethyl-5-aminovalerate	14274897	**1.71**	**1.52**	1.13
Phenylpyruvate	997	*0.73*	0.87	*0.84*
4-Hydroxyphenylpyruvate	979	*0.85*	0.94	*0.91*
Dopamine 3-*O*-sulfate	122136	**1.76**	1.38	1.28
C-Glycosyltryptophan	10981970	**1.84**	1.26	1.46
3-Hydroxy-2-ethylpropionate	188979	**2.19**	**1.62**	1.35
*N*-Acetylvaline	66789	**1.2**	**1.12**	1.07
Isobutyrylglycine	10855600	1.52	**1.62**	*0.94*
5-Methylthioribose	494	**2.91**	**1.9**	1.53
Cystathionine	439258	**1.33**	0.94	**1.41**
Alpha-ketobutyrate	58	*0.93*	1.18	*0.79*
Cysteine *s*-sulfate	115015	*0.95*	0.96	0.99
Lanthionine	98504	**2.13**	1.15	1.86
*N*-Acetyltaurine	159864	**1.47**	1.14	1.28
Urea	1176	**1.65**	**1.5**	1.1
Citrulline	6262	**1.15**	1.02	1.12
Homocitrulline	9750	**1.97**	**1.18**	1.68
Dimethylarginine (SDMA + ADMA)	9085	**1.3**	1.11	1.17
Trans-4-hydroxyproline	65072	**1.48**	1.2	1.23
*N*-Methylproline	123831	**1.51**	1.24	1.22
*N*,*N*,*N*-Trimethyl-alanylproline betaine (TMAP)	5810	**1.88**	**1.83**	1.03
Creatinine	557	**1.31**	**1.23**	1.07
4-Acetamidobutanoate	588	**2.63**	**1.77**	1.48
1-Methylguanidine	131802901	**7.08**	**3.34**	2.12
Gamma-glutamylhistidine	18189	**1.44**	**1.4**	1.03
Gamma-glutamyl-epsilon-lysine	10111	**1.51**	1.03	**1.46**
Gamma-glutamylcitrulline	7017195	**1.33**	1.18	1.12
Arabitol/xylitol	7015685	**1.61**	**1.29**	1.25
Dodecanedioate (C12-DC)	71464481	**1.18**	1.05	**1.13**
Propionylglycine	12736	**1.37**	**1.38**	1
*N*-Palmitoylglycine	98681	**1.19**	**1.27**	0.93
Picolinoylglycine	151008	1.78	**2.19**	0.82
Docosahexaenoylcarnitine (C22:6)	11788622	*0.87*	0.92	*0.94*
2*S*,3*R*-Dihydroxybutyrate	97783	**1.27**	1.24	1.02
2*R*,3*R*-Dihydroxybutyrate	10964471	1.43	**1.54**	0.93
*N*-Myristoyltaurine	13120901	**1.41**	**1.5**	0.94
*N*-Oleoyltaurine	3810823	**1.46**	**1.77**	0.82
*N*-Stearoyltaurine	6437033	**1.27**	1.1	1.16
*N*-Palmitoyltaurine	168274	**1.45**	**1.32**	1.1
*N*-Oleoylserine	2245940	1.25	**1.46**	0.86
1-Palmitoyl-2-oleoyl-GPI (16:0/18:1)	892	1.14	**1.39**	0.82
1-(1-Enyl-palmitoyl)-2-linoleoyl-GPC (P-16:0/18:2)	10917802	**1.55**	1.41	**1.1**
Ceramide (d16:1/24:1, d18:1/22:1)	3081085	**1.62**	1.3	1.25
7-Alpha-hydroxy-3-oxo-4-cholestenoate (7-Hoca)	6675	*0.71*	0.91	*0.78*
Taurochenodeoxycholic acid 3-sulfate	65095	*0.1*	0.08	1.22
*N*1-Methylinosine	203	**1.73**	**1.39**	1.24
Allantoic acid	70765	**3.16**	**2.34**	1.35
1-Methylhypoxanthine	78821	**2.04**	**1.66**	1.23
1-Methyladenine	161466	**1.71**	**1.49**	1.15
*N*6-Carbamoylthreonyladenosine	15047	**1.55**	**1.24**	1.25
Pseudouridine	94312	**1.57**	**1.23**	1.28
5,6-Dihydrouridine	171198	**1.61**	**1.27**	1.27
3-(3-Amino-3-carboxypropyl) uridine	440055	**1.49**	1.15	**1.29**
5-Methyl-2′-deoxycytidine	86484	0.9	*0.81*	**1.11**
Ascorbic acid 2-sulfate	11425365	**1.78**	**1.59**	1.13
Ascorbic acid 3-sulfate	99779	**1.95**	**1.72**	1.13
2-*O*-Methylascorbic acid	151152	**2.09**	**1.51**	1.38
Threonate	638015	**1.43**	**1.23**	1.16
Retinal	464	*0.94*	1.1	*0.86*
Maltol sulfate	5406157	**1.1**	0.55	2.01
Genistein sulfate	222285	*0.69*	0.68	1.02
Erythritol	321710	**2.22**	**1.52**	1.47
Indolin-2-one	444305	**2.15**	**1.62**	1.33
4-Allylphenol sulfate	126041	**1.41**	1.12	**1.26**
Ectoine	6049	**3.14**	**2.35**	1.33
EDTA	74483	0.98	*0.9*	**1.09**
Perfluorooctanesulfonate (PFOS)	65249	**1.21**	1.06	1.14

^a^Values are ratios of means between groups (i.e., RD/Healthy is concentration in cats with renal disease/concentration in cats with healthy kidneys; CaOx/Healthy is concentration in cats with CaOx stones/concentration in cats with healthy kidneys; RD/CaOx is concentration in cats with renal disease/concentration in cats with CaOx stones. Values in bold are above 1 (p < 0.05). Values in italics are lower than 1 (p < 0.05)

## Results

### Classification of cats

**Fig. 1:**
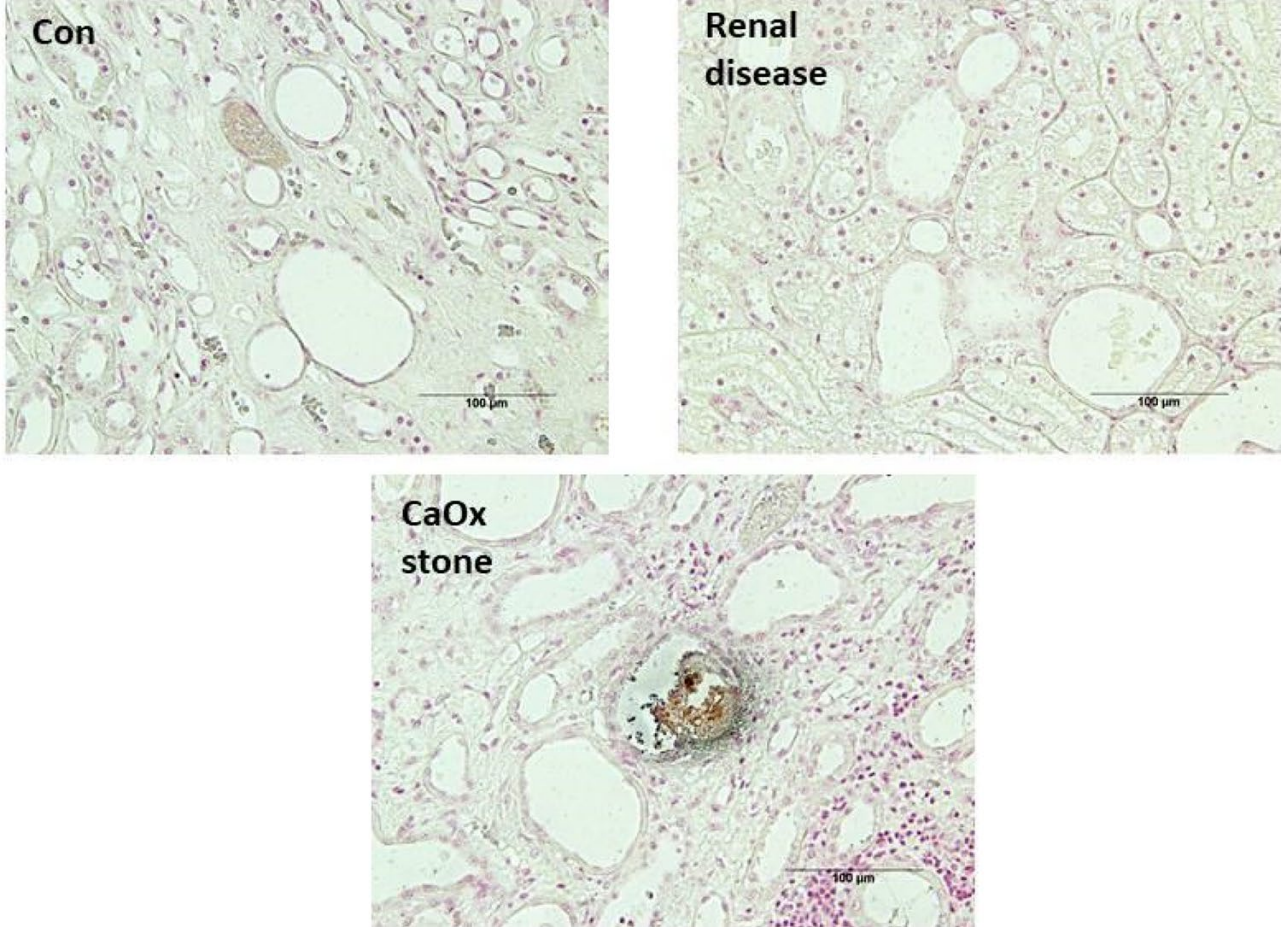
Renal sections stained with Pizzolato stain for detecting CaOx crystals

All cats were placed in their treatment classification after the end of life. Cats were assigned to the CaOx stone forming group by the presence of stones in the kidney that were analyzed as CaOx stones. Cats were assigned to the renal group by evaluation of the kidney, and concentrations of SDMA, creatinine, and uSG. A cat was assigned to the renal disease group if SDMA was over 18 µg/dl, creatinine over 1.6 mg/dl, uSG under 1.030 or had structural signs of renal disease. Cats with confirmed CaOx stones were not made available for the RD group. Healthy kidney cats had no signs of stones or RD throughout life or at death. Calcium Oxalate stone formation was also confirmed through histology, as shown in Fig. 1.

### Plasma metabolomics

There were a significant number of metabolites that increased concentrations in RD and CaOx cats when compared to cats with healthy kidneys. Most of these responded similarly (with an elevated circulating concentrations in the RD and CaOx cats) with the RD cats usually having a more pronounced response. The ten analytes with decreased concentrations in the RD cats (as compared to the healthy cats) were never changed in a significant way in the CaOx cats. This then resulted in eight of the analytes that differed between the CaOx and RD cats being comparatively elevated in the RD cats and seven being comparatively elevated in the CaOxo cats.

### Body composition

At the end of life, cats with CaOx stones had less lean than healthy controls, while cats with RD were not different from either group. During adult life, cats with CaOx stones had a lower fat perentage and mass than cats with RD, healthy controls were not different from either group (Table 3).

**Table 3:** Effect of renal disease or calcium oxalate stone formation on body composition during adult years (> 1 and < 8) and end of life. Values are LSMeans ± standard errors

Variable	Healthy kidneys	Renal disease	CaOx stone formers
Lean (g adult)	3812 ± 111	3635 ± 154	3628 ± 154
Lean (g end of life)	3522 ± 123^a^	3127 ± 162^a,b^	3051 ± 156^b^
Fat (g adult)	1008 ± 101^a,b^	1243 ± 141^a^	764 ± 114^b^
Fat (g end of life)	833 ± 105	931 ± 139	805 ± 134
Bone (g adult)	132 ± 5.2	138 ± 7.3	133 ± 6.0
Bone (g end of life)	130 ± 4.7	130 ± 6.2	131 ± 5.9
Fat % (adult)	19.9 ± 1.7^a,b^	24.4 ± 2.4^a^	16.4 ± 1.9^b^
Fat % (end of life)	18.5 ± 1.9	21.6 ± 2.4	18.7 ± 2.3
Total mass (g adult)	4954 ± 157	5017 ± 219	4525 ± 178
Total mass (g end of life)	4485 ± 187	4179 ± 245	3971 ± 238

^a,b^Means with different superscripts in the same row are different (p < 0.05)

### Specific blood and urine response variables

**Fig. 2:**
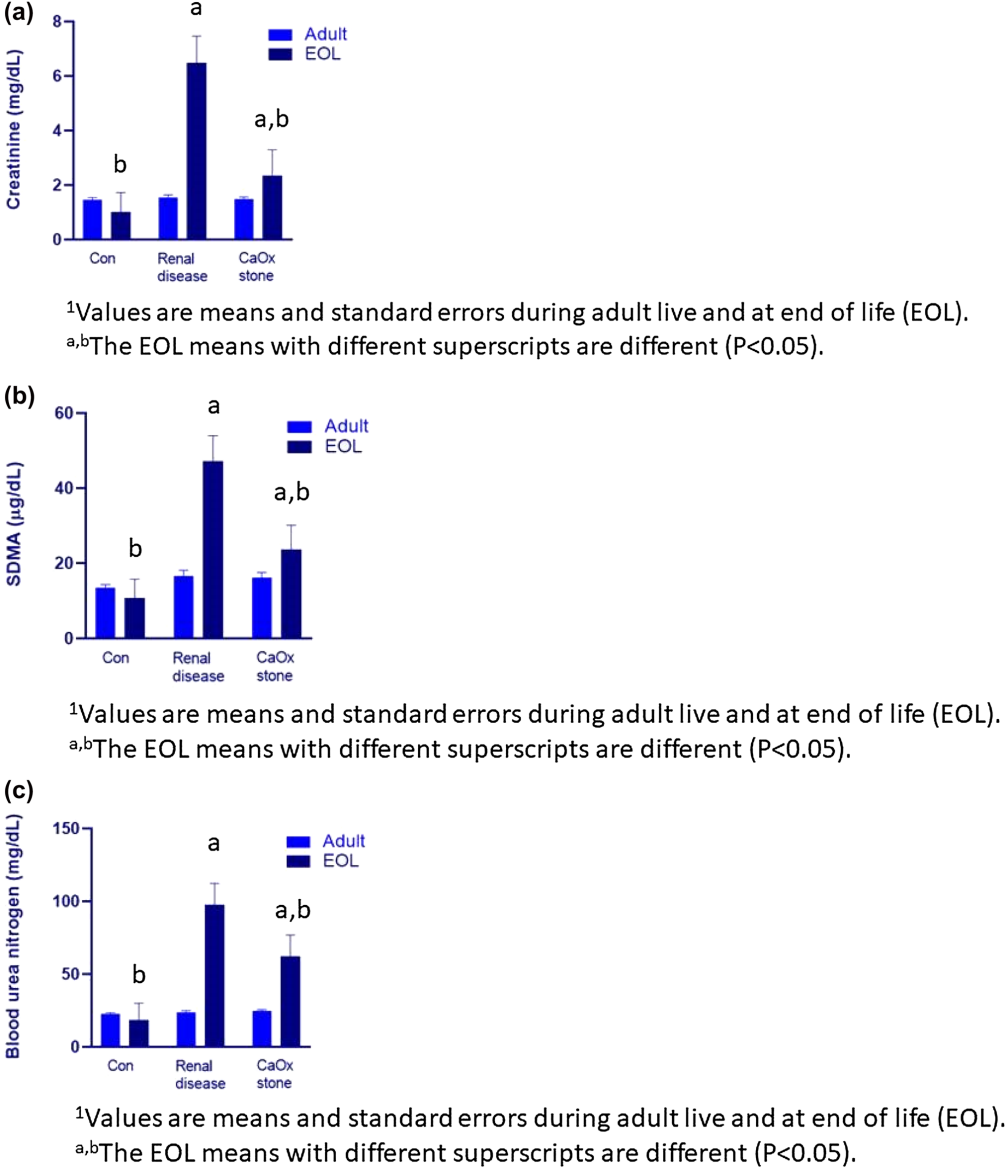
Circulating concentrations of creatinine, SDMA, and urea at the end of life (EOL) of cats with healthy kidneys (Con), renal disease, or CaOx stones

At the end of life, the cats with RD had a lower uSG, as well as higher circulating concentrations of SDMA, creatinine, and urea (Fig. 2). Urine specific gravity was reduced and circulating phophosphorus concentrations were increased when cats with RD were compared to the healthy controls (Table 3). Cats with CaOx stones were not different from the other groups in circulating concentrations, but were different from both groups in uSG (Table 4). There was an effect of gender on creatinine at the end of life (with males having a higher value than females). However, this is likely due to the positive relationship between lean body mass and circulating creatinine as there was no effect of gender when lean was used as a covariant. Gender or lean body mass had no effect on SDMA concentrations. Neutrophils, lymphocytes, and the neutrophil to lymphocyte ratio (NLR) were not different in the cat’s adult years (NLR is an important measure of systemic inflammation). At the end of life, cats with RD had a greater percentage of neutrophils than cats that developed CaOx stones, with healthy controls being not different from either group. At the end of life, the cats which formed CaOx stones had a higher lymphocyte percentage than healthy controls or cats with RD. Cats with RD had a higher NLR ratio at the end of life than healthy controls, with the CaOx forming cats not different from either group (Table 4).

**Table 4:** Effect of renal disease or calcium oxalate stone formation on blood and urine characteristics during adult years (> 1 and < 8) and at end of life. Values are LSMeans ± standard errors

Variable	Adult reference range^d^	Healthy kidneys	Renal disease	CaOx stone formers
Albumin (mg/dl adult)	2.7–3.8	3.19 ± 0.05	3.22 ± 0.07	3.32 ± 0.07
Albumin (mg/dl end of life)		3.04 ± 0.10	3.01 ± 0.13	3.21 ± 0.13
Calcium (mg/dl adult)	8.9–10.9	9.67 ± 0.09	9.79 ± 0.12	9.86 ± 0.11
Calcium (mg/dl end of life)		9.52 ± 0.21	9.55 ± 0.29	9.83 ± 0.28
Phosphorus (mg/dl adult)	3.2–5.4	4.22 ± 0.10	4.30 ± 0.13	4.20 ± 0.13
Phosphorus (mg/dl end of life)		4.09 ± 0.65^b^	6.54 ± 0.89^a^	6.06 ± 0.86^a,b^
Neutrophil % (adult)	Not established	61.5 ± 1.71	60.2 ± 2.24	61.6 ± 2.15
Neutrophil % (end of life)		74.3 ± 2.75^a,b^	80.6 ± 3.53^a^	67.5 ± 3.52^b^
Lymphocyte % (adult)	Not established	28.4 ± 1.80	29.7 ± 2.36	28.8 ± 2.26
Lymphocyte % (end of life)		16.1 ± 2.27^b^	12.8 ± 2.90^b^	24.4 ± 2.90^a^
NLR^e^ (adult)	Not established	2.78 ± 0.35	2.98 ± 0.46	3.06 ± 0.44
NLR^e^ (end of life)		5.56 ± 1.40^b^	10.7 ± 1.93^a^	6.04 ± 1.84^a,b^
Urine specific gravity (adult)	Not established	1.052 ± 0.002	1.047 ± 0.002	1.047 ± 0.002
Urine specific gravity (end of life)		1.047 ± 0.004^a^	1.011 ± 0.005^b^	1.032 ± 0.004^c^

^a,b,c^Means with different superscripts in the same row are different (p < 0.05)

^d^Reference ranges for the research colony where the cats lived

^e^Neutrophil to Lymphocyte ratio

## Discussion

There was a general increase in concentrations of those metabolites dependent on renal clearance in both the RD and CaOx cats (e.g., urea, creatinine, dimethylarginines, pseudouridine). This was shown in that there was an elevation in both cats forming CaOx and RD cats as compared to those with healthy kidneys while there was not an observed difference in creatinine, methylated arginines, pseudouridine or urea between RD and CaOx forming cats. These changes associated with renal function were expected while the other metabolite changes allow for a deeper understanding of these diseases and possible changes in the nutrition to aid in their management.

These data show the expected increase in creatinine as a result of the renal function decline. In a recent review (Kovarikova, 2018), the relationship between creatinine and renal function was elucidated. Increasing lean body mass, and therefore increased creatine, was associated with increased circulating creatinine concentrations (Hall et al., 2014). This explains the gender effect as males, which had higher lean body mass, would be expected to have higher creatinine concentrations. In this study creatinine was highest in the cats with RD, intermediate in the cats with CaOx stones and lowest in cats with healthy kidneys. These changes were not the result of changing lean *body mass*. These data support the conclusion that some obstruction resulted in renal function decline in the cats with CaOx stones. However, this loss of renal function as a group did not equal the function loss of the cats with RD.

The cats with RD also had increased circulating SDMA concentrations as compared to healthy controls, while cats with CaOx stones were intermediate between these groups. The circulating concentrations of SDMA was elevated in cats with CaOx stones as compared to healthy controls (Hall et al., 2017b), but it to our knowledge has not been shown in comparison to cats with RD. Because changes in lean body mass did not account for changes in circulating concentrations of SDMA, there is an improved understanding of renal function through the use of circulating SDMA concentration as compared to circulating creatinine. The lower threshold in renal decline associated with increasing circulating SDMA may contribute to the elevation of circulating SDMA being observed before the elevation of creatinine in cats (Hall et al, 2014).

Urinary specific gravity was different between all three groups at the time of death. This again supports the conclusion that although there was renal decline associated with CaOx stones, although it was not as severe in the RD group. Increasing solute load, specifically urinary calcium, is associated with an increased risk of calcium oxalate stones (Bartges, 2016). However, the pre-disease adult concentrations of solutes, as shown by uSG was not different between groups suggesting that this was not the stimulus for stone formation in these cats.

Phosphorus concentrations were elevated with RD as compared to healthy controls. Elevated circulating phosphorus is a significant risk factor for continued renal decline and death and a direct result of renal decline (Laflamme et al, 2020). Increased circulating calcium concentrations have also been associated with renal decline (Tang et al, 2021) in phosphate restricted foods. The lack of an increase in circulating calcium concentrations (there was a numeric decline) in this study in the cats with RD may be because the cats when diagnosed were transitioned to a different food designed to aid in the management of renal disease than that fed to the cats where circulating calcium concentration increased.

There were metabolomic changes resulting in decreased concentrations in the RD cats while remaining unchanged in the CaOx stone forming cats. The metabolite *N*-acetylasparagine was reduced in RD cats while being unchanged in the healthy controls or cats with CaOx stones. This elevation has previously been reported in humans (Boccard, et al., 2021) and may be the result of a change in the toxification method associated with RD (Sekula, et al., 2016). The relative reduction in phenylpyruvate in RD cats is similar to what has been reported in humans (Kopple, 2007) and may be the result of impaired phenylalanine hydroxylase activity resulting in a reduced phenylalanine to tyrosine conversion (Tourian et al., 1969). There was a reduction in the circulating concentrations of alpha-ketobutyrate with increased concentrations of cystathionine in the RD cats and not in the CaOx cats. This is likely the direct result of homocysteine being irreversibly degraded through the transsulfuration pathway (condensing homocysteine and serine to form cystathionine which is split into alpha-ketobutyrate and cysteine). This could have a significant effect on health as alpha ketobutyrate has been reported to have protective effects in vitro in neuronal cells when stressed with hydrogen peroxide (Jacewicz et al., 2014). These changes support the conclusion that homocysteine degradation is disrupted in cats with RD. Because high circulating concentrations of homocysteine are known to be risk factors in RD in humans (Ostrakhovitch & Tabibzadeh, 2015) and that betaine has been shown to reduce homocysteine and increase methionine in cats (Jewell et al., 2021; Verbrugghe & Bakovic, 2013) these results suggest that dietary betaine could be a benefit to aid in the management of RD in cats. These data then provide understanding for the positive effect of a food containing increased dietary betaine on lean body mass in cats with RD (Ephraim-Gebreselassie et al., 2018) as well as the reduction in oxidized glutathione when dietary betaine was combined with short chain fructo-oligosaccharide (Hall et al., 2020).

The metabolite 1-palmitoyl-GPI, was elevated in the cats with CaOx stones both as compared to cats with RD and cats with healthy kidneys. The elevation of 1-palmitoyl-GPI has been associated with an increased inflammatory condition in humans where it was correlated to the pro-inflammatory cytokine IL-8 (Gao et al., 2021) and was increased in the inflammation associated with periodontitis in mice (Ilievski et al, 2016). Increased concentrations of inflammatory glycosylphosphatidylinositols were observed in the cats eating the more inflammatory foods as compared to initial values or those cats eating a less inflammatory (high fish oil) food (Jewell & Jackson, 2020). It is an intriguing basis for further research to better understand if dietary changes which change the microbiota in cats with CaOx stones would aid in the management of this disease. The increased inflammatory markers in cats with CaOx stones may explain the benefit of a food with a reduced inflammation fatty acid profile in cats with CaOx stones (Hall et al, 2017a, 2017b).

The higher NLR in the cats with RD is likely a function of increased inflammation at end of life. This ratio has been associated with increased inflammation in both people (McMillan, 2009) and cats (Neumann, 2021). This change at the end of life shows the inflammatory whole-body response happening in RD. Although CaOx stones are known to have an inflammatory component (O’kell et al, 2017), this was not reflected in a significant elevation of the NLR. This could result from a more localized inflammation in the cats with CaOx stones compared to those with RD and account for the changing inflammatory metabolomics. This lymphopenia observed in the RD cats was also observed by Kralova-Kovarikova et al. (2016) who found that the reduction was negatively correlated to circulating concentrations of creatinine and urea and resulted in immunosuppression. The reduced immune response was shown by a reduced proliferation in unstimulated cells or cells stimulated with phytohaemaglutinin, concanavalin A, or pokeweed mitogen. The reduced immune response associated with the reduction in NLR may be a significant target for enhancing the length and quality of life in cats with RD.

At the end of life there was a reduction in body lean in the cats with CaOx stones which may have resulted from reduced food intake during the last phase of life. The reduced body fat (and fat percentage) during adult life with body lean being unchanged (similar to healthy controls) suggests a partitioning of available energy away from body fat. It has been reported that lean is preferentially maintained over body fat in cases of reduced food intake (Floerchinger et al, 2015). Although that research was done with food designed to aid in the management of weight loss, it may be that cats with limited calories during life preferentially support body lean, and this is what is observed in the CaOx forming cats body composition.

## Conclusion

Cats with both RD and CaOx stones have metabolomic signs of renal disease and specific metabolomic changes which both give insight into successful management and targets for influence to further aid in the management of these diseases.

## Data Availability

Data are available at Metabolomic Workbench: Metabolomic changes in cats with renal disease and calcium oxalate uroliths.
